# Supplementary material for: Immunophenotype of Measurable Residual Blast Cells as an Additional Prognostic Factor in Adults with B-Cell Acute Lymphoblastic Leukemia
Source: Diagnostics (Basel). 2022 Dec 21;13(1):21. doi: 10.3390/diagnostics13010021 (PMC9818326; doi:10.3390/diagnostics13010021)
Supplement: Supplementary file 1 [file diagnostics-13-00021-s001.zip › diagnostics-1999750-supplementary.pdf]

Supplemental materials

In the period from 2016 to 2019, we used a 2-tube 6-color panel of monoclonal antibodies, and from 2019 to 2021 we used 10-color panels. Ten-color panel No. 1 of **Table S1** included a ready-made mixture of seven antibody conjugates DURAClone RE ALB (Beckman Coulter, France), including antibodies against CD10, CD19, CD20, CD34, CD38, CD45 and CD58. Antibodies against antigens CD24 (clone ML5), CD22 (clone S-HCL-1), as well as Syto41 (Invitrogen) were additionally added to this mixture.

Table S1. Monoclonal antibody panels used in the study

laser, nm	Blue, 488					Red, 633			Violet, 405		
**Six-color panel**											
fluorochrome	FITC	PE	-	PerCP / PerCP-Cy5.5	PE-Cy7	APC	-	APC-Cy7 / APC-H7	-	-	-
Tube 1 (antigen (clone))	CD38 (HIT2)	CD10 (HI10a)	-	CD45 (2D1)	CD34 (8G12)	CD19 (SJ25C1)	-	CD20 (L27)	-	-	-
Tube 2 (antigen (clone))	CD58 (AICD58 or 1C3)	CD10 (HI10a)	-	CD38 (HIT2)	CD34 (8G12)	CD19 (SJ25C1)	-	CD20 (L27)	-	-	-
**Ten-color panel No. 1**											
fluorochrome	FITC	PE	ECD	PC5.5	PC7	APC	APC-A700	APC-A750	-	KrO	-
antigen (clone)	CD58 (AICD58)	CD24 (ALB9)	CD34 (581)	CD10 (ALB1)	CD19 (J3-119)	CD22 (S-HCL-1)	CD38 (LS198-4-3)	CD20 (B9E9)	Syto41	CD45 (J33)	-
**Ten-color panel No. 2**											
fluorochrome	FITC	PE	PE-Dazzle 594	-	PE-Cy7	APC	APC-R700	APC-Fire750	BV421	-	BV650
antigen (clone)	CD58 (AICD58)	CD10 (Hi10a)	CD34 (581)	7-AAD	CD19 (SJ25C1)	CD22 (S-HCL-1)	CD38 (HIT2)	CD45 (Hi30)	CD24 (ML5)	-	CD20 (2H7)

7-AAD – 7-aminoactinomycin D, FITC – fluorescein isothiocyanate, PE – phycoerythrin, ECD – energy coupled dye, PE-Dazzle594 – phycoerythrin Dazzle594, PC5.5 – R Phycoerythrin-Cyanine 5.5, PerCP – peridinin chlorophyll protein, PerCP-Cy5.5 – peridinin chlorophyll protein complex-cyanine 5.5, PE-Cy7 – Phycoerythrin-Cyanine 7, PC7 – R Phycoerythrin-Cyanine 7, APC – allophycocyanine, APC-R700 – allophycocyanine R700, APC-A700 – Allophycocyanin Alexa Fluor 700, APC-Fire750 – allophycocyanine Fire750, APC-Cy7 – allophycocyanine-cyanine 7, APC-A750 – Allophycocyanin Alexa Fluor 750, KrO – Krome Orange, BV – brilliant violet.
